# Comparative Predictive Utility of MM-SES-CD and SES-CD for Week 52 Endoscopic Remission in the Ileum and Colon in Crohn’s Disease

**DOI:** 10.1093/ibd/izaf124

**Published:** 2025-06-16

**Authors:** Emily C L Wong, Parambir S Dulai, John K Marshall, Vipul Jairath, Walter Reinisch, Neeraj Narula

**Affiliations:** Department of Medicine (Division of Gastroenterology) and Farncombe Family Digestive Health Research Institute, McMaster University, Hamilton, ON, Canada; Division of Gastroenterology, Northwestern University, Chicago, IL, USA; Department of Medicine (Division of Gastroenterology) and Farncombe Family Digestive Health Research Institute, McMaster University, Hamilton, ON, Canada; Department of Medicine, Division of Gastroenterology, Western University, London, ON, Canada; Department of Internal Medicine III, Division of Gastroenterology and Hepatology, Medical University of Vienna, Währinger Gürtel 18-20, Vienna, Austria; Department of Medicine (Division of Gastroenterology) and Farncombe Family Digestive Health Research Institute, McMaster University, Hamilton, ON, Canada

**Keywords:** Crohn’s disease, SES-CD, MM-SES-CD

## Abstract

**Background:**

The Simple Endoscopic Score for Crohn’s disease (SES-CD) and Modified Multiplier of the SES-CD (MM-SES-CD) are endoscopic scoring systems used to assess disease severity and response to therapy in CD. This study evaluates their utility at baseline in predicting endoscopic remission (ER) of the ileum and colon at week 52.

**Methods:**

This post-hoc analysis of 4 clinical trials (CT-P13, UNITI, EXTEND, and SEAVUE) compared baseline scores and ER outcomes at week 52 using the SES-CD and MM-SES-CD. The area under the receiver operating characteristic curve (AUC) for each scoring system’s ability to predict week 52 ER defined by various thresholds was compared.

**Results:**

A total of 667 patients were included in this analysis. At baseline, the median SES-CD score was 8.5 (IQR 5.0–15.7), and the median MM-SES-CD score was 34.8 (IQR 27.5–50.0). MM-SES-CD demonstrated consistently higher AUC values compared to SES-CD across most endpoints in ileal and colonic disease involvement. Among 519 patients with any disease in the ileum, baseline MM-SES-CD demonstrated significantly better predictive accuracy than SES-CD for week 52 SES-CD < 3 [AUC 0.75 (95% CI: 0.71–0.80) vs. 0.64 (95% CI: 0.59–0.70), *P* = .031]. Similar findings were observed for other definitions of ER. Among 552 participants with colonic disease involvement, baseline MM-SES-CD also demonstrated significantly greater accuracy for predicting week 52 SES-CD < 3 [AUC 0.78 (95% CI: 0.73–0.82) vs. 0.62 (95% CI: 0.57–0.66), *P* = .002], with similar findings across other definitions of ER assessed. Similar trends were observed across isolated ileal, ileocolonic, and isolated colonic disease.

**Conclusion:**

The baseline MM-SES-CD demonstrated superior predictive ability for week 52 ER compared to SES-CD across the ileum and colon. These findings support its utility in clinical trials and in routine practice for predicting long-term treatment response.

**Key Messages**

**What is already known?**

- The Simple Endoscopic Score for Crohn’s Disease (SES-CD) is widely used in clinical trials to assess disease severity.
- The Modified Multiplier SES-CD (MM-SES-CD) was developed to improve predictive accuracy by adjusting the weight of certain endoscopic parameters.
- Prior studies suggest MM-SES-CD offers improved differentiation of treatment effects and better prediction of endoscopic remission (ER) at 1 year.

**What is new here?**

- This study directly compares MM-SES-CD and SES-CD for predicting week 52 endoscopic remission across the ileum and colon.
- MM-SES-CD consistently outperformed SES-CD in predicting ER across various definitions and disease locations.
- The superior accuracy of MM-SES-CD was particularly evident in distinguishing ileal and colonic healing patterns.

**How can this study help patient care?**

- More accurate prediction of long-term ER using MM-SES-CD can guide treatment decisions, potentially reducing premature therapy changes.
- MM-SES-CD may help better identify responders to therapy, supporting its integration into clinical trials and routine practice.
- Improved endoscopic assessment with MM-SES-CD could enhance disease monitoring and help tailor treatment strategies based on disease location.

## Introduction

Crohn’s disease (CD) is a chronic inflammatory disorder of the gastrointestinal tract, characterized by mucosal ulcerations that can lead to permanent intestinal damage.^1^ Evaluating treatment effectiveness is essential for improving patient outcomes and designing robust clinical trials. The Simple Endoscopic Score for Crohn’s Disease (SES-CD) is a widely used tool in clinical trials to assess endoscopic disease severity.^2^ However, the SES-CD has faced criticism for assigning equal weight to all parameters, which may reduce its prognostic value.

Recently, the Modified Multiplier of the SES-CD (MM-SES-CD) has been introduced as a refined approach to measuring endoscopic disease severity. This method adjusts the weight of individual SES-CD parameters based on its prognostic significance for achieving endoscopic remission (ER) at 1 year.^3^ Compared to the SES-CD, the MM-SES-CD has shown improved accuracy in predicting ER at one year. The MM-SES-CD shed light on the importance of specific characteristics of ulcers and their locations when assessing the likelihood of healing. For instance, compared to other locations, large ileal ulcers were more predictive of the inability to achieve ER.^4^ Furthermore, the utility of the MM-SES-CD in discriminating treatment effects in clinical trials has been demonstrated. In a post-hoc analysis of the EXTEND clinical trial, the MM-SES-CD demonstrated improved discrimination between treatment and placebo with better predictive accuracy for 1-year ER compared to the SES-CD. The nuanced approach of the MM-SES-CD allows for the detection of smaller yet clinically relevant differences, which may lead to false negatives in clinical trials. This may result in some clinicians prematurely switching therapy, falsely concluding that no significant improvement is observed with SES-CD despite experiencing clinically relevant improvement, which might be captured with the MM-SES-CD.

Achievement of ER at 1 year is a key indicator of favorable long-term outcomes, yet limited data exist on whether the MM-SES-CD’s prognostic superiority over the SES-CD applies equally across the ileum and colon. Since the ileum and colon may exhibit differences in healing patterns and response to therapy, understanding the regional accuracy of these scoring systems is essential for refining their use in clinical trials and guiding treatment decisions. The aim of this post-hoc analysis is to compare the predictive ability of baseline MM-SES-CD and SES-CD in the ileum and colon on 1-year ER.

## Methods

### Study Design

This was a post-hoc analysis of individual participant-level data from 4 clinical trial programs, including the UNITI studies (ClinicalTrials.gov identifiers NCT01369329, NCT01369342, NCT01369355), EXTEND (NCT00348283), CT-P13 (NCT02096861), and SEAVUE (NCT03464136). Data were obtained by permission from Celltrion Inc. for the CT-P13 study, from Abbvie Inc. through Vivli (Protocol # 00010706), and from Janssen Inc. through YODA (Protocol # 2024-0888) for the UNITI and SEAVUE studies.^5–8^ Participants included in the current analysis had a baseline SES-CD score ≥ 3 and confirmed mucosal ulceration. All SES-CD scores were converted into MM-SES-CD scores based on methods described previously.^3^

Details regarding the study design and eligibility criteria for each study have been previously described. Briefly, in UNITI, adult patients with moderate-to-severe Crohn’s disease (CDAI 220–450) were enrolled in induction studies, with UNITI-1 including those with prior TNF antagonist failure and UNITI-2 including those with inadequate response to immunomodulators or glucocorticoids.^6^ Inclusion required objective evidence of inflammation (eg, CRP > 3 mg/L, fecal calprotectin > 250 mg/kg, or endoscopic ulceration). Responders to induction were re-randomized in the 44-week maintenance study (IM-UNITI) to receive ustekinumab every 8 or 12 weeks, or placebo, with a total study duration of 52 weeks. Endoscopy was optional but performed on a subset at baseline, week 8, and week 52, with all readings centralized. Non-responders could receive open-label ustekinumab and participate in the endoscopic sub-study.

In EXTEND, adults with moderate to severely active CD (CDAI 220–450) and mucosal ulceration on colonoscopy were included.^5^ Eligible patients had an inadequate response to conventional therapy or prior TNF antagonist use (excluding primary non-responders). All participants received 4 weeks of adalimumab induction before re-randomization to adalimumab maintenance (40 mg every other week) or placebo. Endoscopies were performed at baseline, week 12, and week 52, with central reading. For this study, we only included participants who were treated with adalimumab continuously for the entire duration of the study.

In CT-P13, patients with moderate-to-severe CD were randomized to one of 4 treatment groups, receiving either CT-P13 or innovator infliximab, with crossover at week 30 (7). Treatments involved infliximab 5 mg/kg infusions at standard induction and maintenance intervals every 8 weeks. The 54-week study included ileo-colonoscopies at baseline and end of study, which were all centrally read.

SEAVUE was an active comparator trial comparing ustekinumab and adalimumab in the treatment of moderate-severely active CD (CDAI 220-450).^8^ Patients were biologic-naïve and had evidence of mucosal ulcerations at baseline. Eligible patients were randomized to receive 6 mg/kg ustekinumab intravenously and 90 mg subcutaneously every 8 weeks or 160 mg adalimumab subcutaneously followed by 80 mg at week 2 and 40 mg every 2 weeks thereafter to week 56. Endoscopies were performed at baseline and week 52 and were centrally read.

For this analysis, we only included participants with SES-CD ≥ 3 and confirmed mucosal ulceration who were treated continuously for 1 year with biologic therapy. In the UNITI studies, 334 subjects participated in the endoscopy sub-study. Participants who crossed over between ustekinumab and placebo were excluded from the analysis, 80 individuals who received continuous ustekinumab throughout the 52-week period were included. The EXTEND study enrolled 129 patients who underwent baseline endoscopic assessments, of which a total of 64 participants were randomized to receive adalimumab continuously, and all of these patients met the inclusion criteria for this analysis. In the CT-P13 study, of the 220 randomized patients, 141 met the inclusion criteria and had both baseline and week 54 ileocolonoscopy data available for evaluation. From SEAVUE, a total of 382 participants were randomized to ustekinumab or adalimumab with available endoscopic data and were included in the current analysis.

### Outcomes

The primary outcome of this study was week 52 ER, defined as SES-CD < 3. Other definitions of week 52 ER were assessed as secondary outcomes, including SES-CD of 0, < 4, ≤ 4, and reduction from baseline ≥ 50% as well as MM-SES-CD < 22.5, based on previous studies suggesting these targets for ER.^9^

### Statistical Analysis

Descriptive statistics were used to summarize the demographic and baseline characteristics of the study population. Categorical variables were expressed as proportions or percentages and compared using Chi-squared tests, while continuous variables were summarized using means with standard deviations (SD) or medians with interquartile ranges (IQR) and compared using t-tests. Analyses were performed on an intention-to-treat basis. In the ileum analysis, MM-SES-CD scores of the ileum were assessed among participants with isolated ileal or ileocolonic disease. In the colonic analysis, MM-SES-CD scores of the colon were assessed among participants with ileocolonic or colonic disease. Subgroup analyses were performed to explore the predictive value of MM-SES-CD and SES-CD in specific disease locations (isolated ileal, ileocolonic, or colonic only). Specifically, subgroup analyses of patients with ileocolonic disease and baseline SES-CD ≥6 were used to exclude those with mild disease activity.^10–12^ These analyses aimed to identify potential differences in the scoring systems’ sensitivity and accuracy across varying CD phenotypes.

To evaluate the predictive performance of baseline MM-SES-CD and SES-CD in achieving week 52 ER, receiver operating characteristic (ROC) curve analyses were performed, and the area under the curve (AUC) was calculated. The AUC served as a summary measure of the scoring systems’ discriminatory ability to predict ER. AUC values of baseline MM-SES-CD and SES-CD for each outcome were generated and compared. Statistical significance between AUC values was assessed using DeLong’s test, with *P*-values < .05 considered statistically significant. All statistical analyses were conducted using Stata IC version 18.

## Results

### Baseline Characteristics

Details regarding the baseline demographic characteristics of the study population are provided in Table 1. A total of 667 participants were included, of which 160 (24%) had isolated ileal disease, 314 (47.1%) had ileocolonic disease and 193 (28.9%) had a disease in the colon only. The mean age of participants was 36.9 years (SD 12.8), and 50.5% were female. The mean duration CD was 7.0 years (SD 7.7). The median SES-CD score was 8.5 (IQR 5.0–15.7), while the median MM-SES-CD score was 34.8 (IQR 27.5–50.0). The mean CDAI score was 305.7 (SD 60.9). Prior anti-TNF use was reported in 8.5% of patients, and 39% were on concomitant corticosteroids. A greater proportion of participants in the CT-P13 study had colonic disease compared to other studies included. Otherwise, demographic characteristics were similar across all studies included.

**Table 1.** Baseline demographics of the study population.

	Overall (*n* = 667)	CT-P13 (*n* = 141)	UNITI (*n* = 80)	EXTEND (*n* = 64)	SEAVUE (*n* = 382)
Age, mean (SD)	36.9 (12.8)	35.3 (12.7)	38.1 (13.1)	37.1 (11.8)	37.2 (13.1)
Female, *n* (%)	337 (50.5)	63 (44.7)	33 (41.3)	40 (62.5)	201 (52.6)
CD duration, mean (SD)	7.0 (7.7)	6.3 (3.8)	10.1 (8.5)	10.1 (8.1)	5.6 (7.9)
CD location, *n* (%)					
Isolated ileal	160 (24.0)	28 (19.9)	16 (20.0)	24 (37.5)	92 (24.1)
Colon	193 (28.9)	93 (66.0)	20 (25.0)	20 (31.3)	60 (15.7)
Ileocolonic	314 (47.1)	20 (14.2)	44 (55.0)	20 (31.3)	230 (60.2)
CDAI score, mean (SD)	305.7 (60.9)	296.4 (54.7)	321.5 (57.4)	319.9 (70.1)	301.0 (60.2)
SES-CD score, median (IQR)	8.5 (5.0–15.7)	9.7 (5.7–15.7)	8.8 (5.8–13.3)	9.5 (5.5–13.2)	7.6 (5.0–13.0)
MM-SES-CD score, median (IQR)	34.8 (27.5–50.0)	32.7 (27.5–38.4)	35.4 (29.6–34.2)	37.5 (30.1–50.0)	34.5 (28.5–39.5)
Prior anti-TNF use, *n* (%)	57 (8.5)	0	30 (37.5)	27 (42.2)	0
Concomitant corticosteroid use, n (%)	260 (39.0)	45 (31.9)	37 (46.3)	14 (21.9)	164 (42.9)

### Predictive Accuracy for Week 52 Endoscopic Remission in the Ileum and Colon

Table 2 demonstrates the comparative performance of baseline MM-SES-CD and SES-CD scores with involvement in the ileum and colon for predicting various definitions of week 52 ER. Among the 474 patients with disease in the ileum (including 314 participants with ileocolonic disease and 160 participants with isolated ileal disease), baseline MM-SES-CD demonstrated significantly better predictive accuracy than SES-CD for week 52 ileal SES-CD < 3 [AUC 0.75 (95% CI: 0.71–0.80) vs. 0.64 (95% CI: 0.59–0.70), *P* = .031]. Similar findings were observed for other definitions of ER, including SES-CD of 0 [AUC 0.75 (95% CI: 0.69–0.78) vs. 0.65 (95% CI: 0.60–0.69), *P* = .029], SES-CD < 4 [AUC 0.74 (95% CI: 0.69–0.78) vs. 0.60 (95% CI: 0.55–0.66), *P* = .031], SES-CD ≤ 4 [AUC 0.73 (95% CI: 0.67–0.79) vs. 0.64 (95% CI: 0.59–0.69), *P* = .042], SES-CD reduction ≥ 50% from baseline [AUC 0.72 (95% CI: 0.67–0.77) vs. 0.62 (95% CI: 0.56–0.68), *P* = .033], MM-SES-CD < 22.5 [AUC 0.68 (95% CI: 0.64–0.72) vs. 0.61 (95% CI: 0.56–0.66), *P* = .015], and MM-SES-CD < 22.5 among those with MM-SES-CD ≥ 22.5 at baseline [AUC 0.70 (95% CI: 0.66–0.74) vs. 0.64 (95% CI: 0.58–0.68), *P* = .004].

**Table 2.** Comparison of baseline MM-SES-CD and SES-CD for predicting week 52 endoscopic remission with colon and ileum involvement.

	Baseline MM-SES-CD AUC (95% CI)	Baseline SES-CD AUC (95% CI)	*P*-value
Ileal disease (isolated ileal or ileocolonic—*n* = 474)			
Week 52 SES-CD < 3	0.75 (0.71–0.80)	0.64 (0.59–0.70)	.031
Week 52 SES-CD of 0	0.74 (0.69–0.78)	0.65 (0.60–0.69)	.029
Week 52 MM-SES-CD < 22.5	0.68 (0.64–0.72)	0.61 (0.56–0.66)	.015
Week 52 MM-SES-CD < 22.5 among those with MM-SES-CD ≥ 22.5 at baseline	0.70 (0.66–0.74)	0.64 (0.58–0.68)	.004
Week 52 SES-CD < 4	0.74 (0.69–0.78)	0.60 (0.55–0.66)	.031
Week 52 SES-CD ≤ 4	0.73 (0.67–0.79)	0.64 (0.59–0.69)	.042
Week 52 SES-CD reduction ≥ 50% from baseline	0.72 (0.67–0.77)	0.62 (0.56–0.68)	.033
Colonic disease (ileocolonic or colonic—*n* = 507)			
Week 52 SES-CD < 3	0.78 (0.73–0.82)	0.62 (0.57–0.66)	.002
Week 52 SES-CD of 0	0.77 (0.73–0.81)	0.62 (0.57–0.66)	.011
Week 52 MM-SES-CD < 22.5	0.73 (0.67–0.78)	0.61 (0.57–0.65)	.005
Week 52 MM-SES-CD < 22.5 among those with MM-SES-CD ≥ 22.5 at baseline	0.74 (0.70–0.78)	0.62 (0.58–0.66)	.003
Week 52 SES-CD < 4	0.78 (0.74–0.82)	0.65 (0.61–0.69)	.032
Week 52 SES-CD ≤ 4	0.78 (0.74–0.82)	0.65 (0.61–0.69)	.031
Week 52 SES-CD reduction ≥ 50% from baseline	0.77 (0.73–0.81)	0.64 (0.59–0.69)	.027

Among the 507 participants with colonic disease (including 314 participants with ileocolonic disease and 193 participants with colonic disease), compared to baseline SES-CD, baseline MM-SES-CD had significantly greater accuracy for predicting week 52 colonic SES-CD < 3 [AUC 0.78 (95% CI: 0.73–0.82) vs. 0.62 (95% CI: 0.57–0.66), *P* = .002], SES-CD of 0 [AUC 0.77 (95% CI: 0.73–0.81) vs. 0.62 (95% CI: 0.57–0.66), *P* = .01], MM-SES-CD < 22.5 [AUC 0.73 (95% CI: 0.67–0.78) vs. 0.61 (95% CI: 0.57–0.65), *P* = .005], MM-SES-CD < 22.5 among those with MM-SES-CD ≥ 22.5 at baseline [AUC 0.74 (95 CI: 0.70–0.78) vs. 0.62 (95% CI: 0.58–0.66), *P* = .003], SES-CD < 4 [AUC 0.78 (95% CI: 0.74–0.82) vs. 0.65 (95% CI: 0.61–-0.69), *P* = .032], SES-CD ≤ 4 [AUC 0.78 (95% CI: 0.74–0.82) vs. 0.65 (95% CI: 0.61–0.69), *P* = .031], and SES-CD reduction ≥ 50% from baseline [AUC 0.77 (95% CI: 0.73–0.81) vs. 0.64 (95% CI: 0.59–0.69), *P* = .027].

### Subgroup Analysis by Disease Location

In the subgroup analysis stratified by disease location, similar findings were observed when the performance of baseline MM-SES-CD and SES-CD were compared among 160 patients with isolated ileal disease (Table 3). Sensitivity analyses were also performed among 114 patients with isolated ileal disease and baseline SES-CD ≥ 4, which demonstrated similar findings. Baseline MM-SES-CD significantly outperformed the SES-CD across all week 52 definitions of ER assessed among 193 patients with the colonic disease and 314 patients with ileocolonic disease. Findings were consistent when analyses were restricted to 246 patients with ileocolonic disease and baseline SES-CD ≥ 6.

**Table 3.** Subgroup analyses comparing baseline MM-SES-CD and SES-CD for predicting week 52 endoscopic remission based on disease location

	Baseline MM-SES-CD AUC (95% CI)	Baseline SES-CD AUC (95% CI)	*P*-value
Isolated Ileal (n = 160)			
Week 52 SES-CD < 3	0.74 (0.64–0.88)	0.65 (0.61–0.89)	.354
Week 52 SES-CD of 0	0.71 (0.62–0.85)	0.62 (0.54–0.72)	.186
Week 52 MM-SES-CD < 22.5	0.70 (0.61–0.80)	0.63 (0.57–0.70)	.248
Week 52 MM-SES-CD < 22.5 among those with MM-SES-CD ≥ 22.5 at baseline	0.68 (0.60–0.75)	0.65 (0.59–0.71)	.602
Week 52 SES-CD < 4	0.72 (0.60–0.89)	0.67 (0.62–0.74)	.463
Week 52 SES-CD ≤ 4	0.72 (0.61–0.89)	0.67 (0.62–0.74)	.411
Week 52 SES-CD reduction ≥ 50% from baseline	0.72 (0.63–0.81)	0.67 (0.62–0.73)	.259
Colonic (n = 193)			
Week 52 SES-CD < 3	0.81 (0.70–0.91)	0.62 (0.55–0.68)	.008
Week 52 SES-CD of 0	0.81 (0.70–0.91)	0.62 (0.54–0.68)	.015
Week 52 MM-SES-CD < 22.5	0.72 (0.65–0.79)	0.60 (0.55–0.66)	.024
Week 52 MM-SES-CD < 22.5 among those with MM-SES-CD ≥ 22.5 at baseline	0.73 (0.65–0.80)	0.61 (0.56–0.65)	.036
Week 52 SES-CD < 4	0.80 (0.72–0.90)	0.63 (0.53–0.73)	.017
Week 52 SES-CD ≤ 4	0.80 (0.72–0.90)	0.63 (0.53–0.73)	.017
Week 52 SES-CD reduction ≥ 50% from baseline	0.78 (0.70–0.87)	0.65 (0.57–0.75)	.024
Ileocolonic (n = 314)			
Week 52 SES-CD < 3	0.77 (0.68–0.86)	0.63 (0.57–0.69)	.042
Week 52 SES-CD of 0	0.77 (0.68–0.86)	0.64 (0.57–0.72)	.175
Week 52 MM-SES-CD < 22.5	0.67 (0.63–0.72)	0.60 (0.53–0.67)	.005
Week 52 MM-SES-CD < 22.5 among those with MM-SES-CD ≥ 22.5 at baseline	0.71 (0.60–0.81)	0.62 (0.56–0.70)	.044
Week 52 SES-CD < 4	0.75 (0.64–0.86)	0.63 (0.52–0.72)	.033
Week 52 SES-CD ≤ 4	0.75 (0.64–0.86)	0.63 (0.52–0.72)	.033
Week 52 SES-CD reduction ≥ 50% from baseline	0.74 (0.64–0.85)	0.62 (0.55–0.71)	.025
Isolated Ileal (*n* = 114 among patients with a baseline SES-CD ≥ 4)			
Week 52 SES-CD < 3	0.71 (0.59–0.84)	0.63 (0.56–0.80)	.342
Week 52 SES-CD of 0	0.68 (0.56–0.80)	0.63 (0.55–0.80)	.426
Week 52 MM-SES-CD < 22.5	0.69 (0.55–0.72)	0.61 (0.52–0.70)	.153
Week 52 MM-SES-CD < 22.5 among those with MM-SES-CD ≥ 22.5 at baseline	0.65 (0.52–0.74)	0.63 (0.56–0.81)	.523
Week 52 SES-CD < 4	0.70 (0.53–0.84)	0.65 (0.57–0.82)	.259
Week 52 SES-CD ≤ 4	0.71 (0.53–0.84)	0.65 (0.57–0.82)	.201
Week 52 SES-CD reduction ≥ 50% from baseline	0.68 (0.55–0.81)	0.62 (0.55–0.71)	.235
Ileocolonic (*n* = 246 among patients with a baseline SES-CD ≥ 6)			
Week 52 SES-CD < 3	0.75 (0.65–0.88)	0.62 (0.53–0.71)	.035
Week 52 SES-CD of 0	0.76 (0.64–0.89)	0.61 (0.52–0.73)	.023
Week 52 MM-SES-CD < 22.5	0.68 (0.58–0.80)	0.60 (0.49–0.72)	.042
Week 52 MM-SES-CD < 22.5 among those with MM-SES-CD ≥ 22.5 at baseline	0.70 (0.62–0.81)	0.63 (0.51–0.76)	.039
Week 52 SES-CD < 4	0.72 (0.64–0.80)	0.64 (0.50–0.77)	.022
Week 52 SES-CD ≤ 4	0.72 (0.64–0.80)	0.64 (0.50–0.77)	.022
Week 52 SES-CD reduction ≥ 50% from baseline	0.73 (0.62–0.85)	0.60 (0.48–0.71)	.045

### Impact of Treatment Received

Univariate analyses were performed to determine if an association between treatment received by participants (infliximab, adalimumab, or ustekinumab) and achievement of SES-CD < 3 in the ileum and colon existed. No significant association between treatment and achievement of one-year SES-CD < 3 in the ileum (*P* = .263) or the colon (*P* = .117) was identified.

## Discussion

In this study, baseline MM-SES-CD consistently outperformed the SES-CD in predicting ER at 1 year across various definitions and disease locations. Notably, we found that the MM-SES-CD had better predictive accuracy in the ileum and colon, with similar findings observed among patients with isolated ileal, ileocolonic, and colonic disease. The MM-SES-CD’s improved accuracy underscores its potential as a more prognostic tool for evaluating endoscopic disease severity in CD, particularly in clinical trials and treatment decision-making.

Among participants with ileal involvement, baseline MM-SES-CD achieved significantly higher AUC values than SES-CD for multiple ER definitions, including SES-CD < 3, SES-CD < 4, and a reduction of SES-CD by ≥ 50%. Similarly, MM-SES-CD demonstrated superior predictive performance in the colon. These findings persisted in subgroup analyses stratified by disease location across isolated ileal, colonic, and ileocolonic diseases. Notably, in patients with baseline SES-CD scores exceeding thresholds for moderate disease, the MM-SES-CD retained its predictive advantage, underscoring its robustness across varying degrees of disease severity.

The MM-SES-CD likely achieves better predictive accuracy due to its incorporation of prognostic weight adjustments for specific parameters, such as the size and location of ulcers. By addressing limitations inherent to the SES-CD, which treats all parameters equally, the MM-SES-CD provides a more nuanced assessment of healing potential. This is especially relevant given the regional heterogeneity of CD, with differing healing patterns and treatment responses between the ileum and colon. Evidence from previous studies have suggested that ulcers in the ileum are the most challenging to heal using biological therapies.^13,14^ Previously, we found that ulcer size and depth have greater prognostic significance compared to the overall degree of endoscopic inflammation, particularly in the ileum and rectum.^15^ Our current analysis builds on these findings by demonstrating that baseline MM-SES-CD of the ileum and colon provides a more accurate reflection of these ulcer characteristics given the differential weighting applied that is absent in the SES-CD, thereby improving its predictive power for long-term ER. Specifically, the incorporation of ulcer size and depth as weighted parameters allows the MM-SES-CD to better capture the clinical significance of these features, particularly in the ileum, where large ulcers are more refractory to therapy. In contrast, superficial and inflammatory lesions in the colon may respond more effectively to biologics. This disparity underscores the importance of tailoring scoring systems to account for regional differences in disease manifestation. By integrating these nuances, the MM-SES-CD provides a more precise prediction of outcomes based on baseline scores. These advancements make the MM-SES-CD a valuable tool in both clinical practice and research, allowing for more targeted interventions and enhanced monitoring of ulcer healing across different disease locations. The improved discriminatory power of the MM-SES-CD could reduce the required sample sizes in clinical trials and allow for more efficient study designs. Furthermore, early prediction of endoscopic remission could allow clinicians to tailor therapy sooner, potentially improving long-term outcomes. However, it is important to note that endoscopic scoring systems like SES-CD, and even MM-SES-CD to some extent, may be less accurate in assessing isolated ileal disease. First, the ileum is more challenging to intubate during colonoscopy, which may lead to an underestimation of the disease burden. Second, inflammation in the ileum often presents with fewer superficial lesions and more deep or transmural features, which may not be fully captured by mucosal-based scoring systems.

Strengths of this study include the use of robust individual patient-level data from several clinical trial programs with centrally or locally read endoscopy. We assessed multiple definitions of ER which enhances the generalizability of findings. However, several limitations are worth noting. While the MM-SES-CD demonstrated superior predictive accuracy, its utility in routine clinical practice may be limited by its complexity at the point of care. However, an MM-SES-CD calculator is available online (https://www.mcmasteribd.com/mm-ses-cd) to facilitate easier calculation of the score. Furthermore, we were only able to assess outcomes at 1 year, and further research is needed to assess the prognostic accuracy of the MM-SES-CD over longer time periods. Lastly, our analysis was underpowered for subgroups analyses (eg, isolated ileal disease). Specifically, the sample size for the isolated ileal subgroup (*n* = 160) provided 60% power to detect the observed difference in AUCs between MM-SES-CD and SES-CD (difference of 0.09). Future studies should focus on larger cohorts of ileal CD to validate our findings. Analyses stratified by specific biologic therapies would have also been underpowered and future studies are encouraged to provide further insights into the MM-SES-CD’s performance across different treatment modalities, including small molecule therapies. There may be other baseline variables that impact the likelihood of achieving 1-year ER, such as treatment received. In our study, univariate analyses did not demonstrate a significant association between treatment received and achievement of ER in the ileum and colon separately. Future studies should also consider the effect of different treatments. These limitations should be addressed by assessing the MM-SES-CD in diverse clinical settings with longer follow-up periods.

In conclusion, the MM-SES-CD represents a significant advancement over the SES-CD for predicting ER in CD, particularly when considering the ileum and colon separately. By incorporating weighted parameters with prognostic relevance, the MM-SES-CD offers a more accurate and practical tool for both clinical trials and routine practice. Its superior predictive ability underscores its potential to enhance clinical decision-making and improve patient outcomes.

## Data Availability

This publication (Vivli protocol #00010706) is based on research using data from Abbvie Inc that has been made available through Vivli, Inc. Vivli has not contributed to or approved, and is not in any way responsible for, the contents of this publication. This study, carried out under YODA Project # 2024-0888, used data obtained from the Yale University Open Data Access Project, which has an agreement with JANSSEN RESEARCH & DEVELOPMENT, L.L.C. The interpretation and reporting of research using this data are solely the responsibility of the authors and does not necessarily represent the official views of the Yale University Open Data Access Project or JANSSEN RESEARCH & DEVELOPMENT, L.L.C.
